# Clinical Impact of Admission Day on Outcomes in Acutely Decompensated Aortic Stenosis: A Nationwide Analysis

**DOI:** 10.3390/jpm14121118

**Published:** 2024-11-25

**Authors:** Nahush Bansal, Eun Seo Kwak, Mohammad Alqadi, Shuhao Qiu, Ragheb Assaly

**Affiliations:** 1 Department of Internal Medicine, The University of Toledo, Toledo, OH 43606, USAshuhao.qiu@utoledo.edu (S.Q.); 2 Department of Pulmonary and Critical Care Medicine, The University of Toledo, Toledo, OH 43606, USA

**Keywords:** aortic stenosis, admission day, weekend effect, mortality, TAVR, transcatheter aortic valve replacement

## Abstract

**Background/Objectives:** Aortic stenosis (AS) is a critical valvular heart disease associated with significant morbidity and mortality if not managed promptly. Previous studies have highlighted the “weekend effect”, where the day of admission impacts outcomes in various cardiac conditions. This study evaluates the impact of weekend versus weekday admissions on outcomes in patients admitted with acutely decompensated aortic stenosis. **Methods:** We conducted a retrospective cohort study using the National Inpatient Sample database, focusing on patients emergently admitted with decompensated aortic stenosis. Patient outcomes were compared between those admitted on weekends (midnight Friday to midnight Sunday) and weekdays. **Results:** Among 94,320 patients included, 4537 (4.81%) were admitted on weekends. Weekend admissions were associated with significantly higher mortality (aOR 1.86; 95% CI 1.27–2.74) and longer hospital stays (mean increase 3.18 days; *p* < 0.001) compared to weekday admissions. Complication rates were also higher on weekends, including cardiogenic shock (aOR 2.1; 95% CI 1.54–2.87; *p* < 0.001), acute kidney injury (aOR 2.48; 95% CI 2.09–2.94; *p* < 0.001), and acute respiratory failure (aOR 2.88; 95% CI 2.38–3.49; *p* < 0.001). Additionally, weekend admissions had lower rates of transcatheter aortic valve replacement (TAVR) (aOR 0.49; 95% CI 0.40–0.62; *p* < 0.01) than weekday admissions. **Conclusions:** Patients with aortic stenosis admitted on weekends face significantly higher mortality, extended hospital stays, and increased complication rates compared to weekday admissions. Transcatheter aortic valve replacement (TAVR) rates were also lower for patients admitted on weekends. The “weekend effect” markedly influences outcomes, underscoring the need for hospital and administrative strategies to mitigate these adverse effects. Implementing standardized protocols and optimizing resource allocation during weekends could potentially reduce mortality and improve patient outcomes, offering a path to more equitable healthcare delivery.

## 1. Introduction

Aortic stenosis is a prevalent valvular disorder, particularly among the elderly, which can lead to significant left ventricular outflow obstruction. The prevalence of aortic stenosis rises with age, with approximately 2 to 9% of individuals over 75 affected by severe aortic stenosis [1,2]. The primary causes include congenital conditions, such as bicuspid aortic valve, calcific degeneration associated with aging, and rheumatic heart disease, with variations seen across different geographical regions. Patients may remain asymptomatic for extended periods but eventually develop symptoms like exertional dyspnea, fatigue, chest pain, and dizziness. Once symptoms manifest, the condition becomes rapidly fatal, and aortic valve replacement (AVR) is strongly indicated [3,4]. Without AVR, the mortality rate in symptomatic aortic stenosis patients is approximately 2% per month, with 75% of patients succumbing within three years [5].

Given the slow, progressive nature of the disease, most patients with aortic stenosis are closely monitored. However, a subset of these patients may get admitted with acute decompensation, potentially leading to life-threatening heart failure, syncope, or angina. In some cases, this may even be the first presentation of previously undiagnosed aortic stenosis. These patients require rapid symptom control, hemodynamic and respiratory support as needed, and prompt management with aortic valve replacement. Transcatheter aortic valve replacement (TAVR) is the preferred treatment for patients with symptomatic aortic stenosis. Urgent TAVR during the same hospitalization has proven to be a feasible and effective option in patients admitted with acutely decompensated aortic stenosis, significantly improving outcomes [6,7].

The timing of hospital admission has been shown to significantly affect both patient and hospital outcomes in various emergent cardiac conditions, with weekend admissions often associated with poorer outcomes [8,9,10,11,12,13]. Contributing factors for this observed “weekend effect” include reduced staffing, higher workload per person, limited familiarity with patients, delayed access to diagnostic testing, and the lower availability of specialized expertise during weekends [11]. This leads to higher mortality, morbidity, and complication rates in weekend admissions. We hypothesized that patients with acutely decompensated aortic stenosis may be particularly vulnerable to this effect, given the urgency of care required.

This study examines the impact of the day of admission on key hospital and patient outcomes, including mortality, resource utilization like length of stay and hospitalization charges, and complication and TAVR rates in those admitted with acutely decompensated aortic stenosis, utilizing data from a large nationwide database.

## 2. Materials and Methods

This is a retrospective cohort study of adult patients hospitalized in 2020 across acute care hospitals in the United States. The analysis was conducted using the National Inpatient Sample (NIS), developed by the Agency for Healthcare Research and Quality (AHRQ). NIS is the largest publicly available all-payer inpatient database, representing nonfederal acute care hospitals nationwide. Hospitals are stratified by ownership, control, bed size, teaching status, urban/rural location, and geographic region. A 20% probability sample of hospitals from each stratum is collected, and all discharges from these hospitals are recorded and weighted to generate national estimates. The 2020 NIS includes data from 49 statewide data organizations, covering 98% of the U.S. population, and provides detailed hospital and patient-level information.

Patients were selected using the International Classification of Diseases, Tenth Revision, Clinical Modification (ICD-10-CM) coding system. ICD-10-CM codes for the principal diagnosis of aortic stenosis were identified, and all adult patients above the age of 18 years emergently admitted with aortic stenosis were included in the study. Patients were excluded if they were younger than 18 years of age or if the admission was elective rather than emergent using the NIS data elements. The patients who underwent surgical aortic valve replacement (SAVR) were also excluded using the ICD-10 codes. The element “AWEEKEND”, which is predefined in the NIS, classifies admissions as occurring either on weekends (12:00 a.m. Saturday to 11:59 p.m. Sunday) or weekdays (12:00 a.m. Monday to 11:59 p.m. Friday). All ICD-10-CM codes used in this study for diagnoses, procedures, and outcomes are listed in the Supplementary Table S1. Ethical approval and informed consent were waived for this study by the University of Toledo ethical board, as this study was conducted using the National inpatient database. This database contains publicly available de-identified data, which makes it impractical to obtain individual consent. 

The primary outcome of this study was in-hospital mortality. Secondary outcomes included length of stay (LOS), total hospital charges, and complications such as cardiogenic shock, acute respiratory failure, and acute kidney injury, as well as the occurrence of valvuloplasty and transcatheter aortic valve replacement (TAVR) procedure. The primary exposure of interest was the day of admission, specifically whether patients were admitted on a weekend versus a weekday.

Data were analyzed using the Software for Statistics and Data Science (STATA/MP 18.0, Stata Corp., College Station, TX, USA). A univariate screen was initially performed to assess different outcomes in aortic stenosis patients. Multivariate logistic regression was then used to adjust for potential confounders, including age, sex, race, median income, patient comorbidities (measured using the Charlson Comorbidity Index), hospital geographic region (Northeast, Midwest, West, or South), hospital bed size, hospital academic status, hospital location (rural or urban), and insurance status. Continuous variables were expressed as means with standard deviations (95% CI), and regression analysis was employed to compare differences between weekday and weekend admissions. The chi-squared test was conducted to compare categorical variables. Throughout the analysis, a two-sided *p* value < 0.05 was considered statistically significant.

## 3. Results

### 3.1. Patient Characteristics

**Figure 1:**
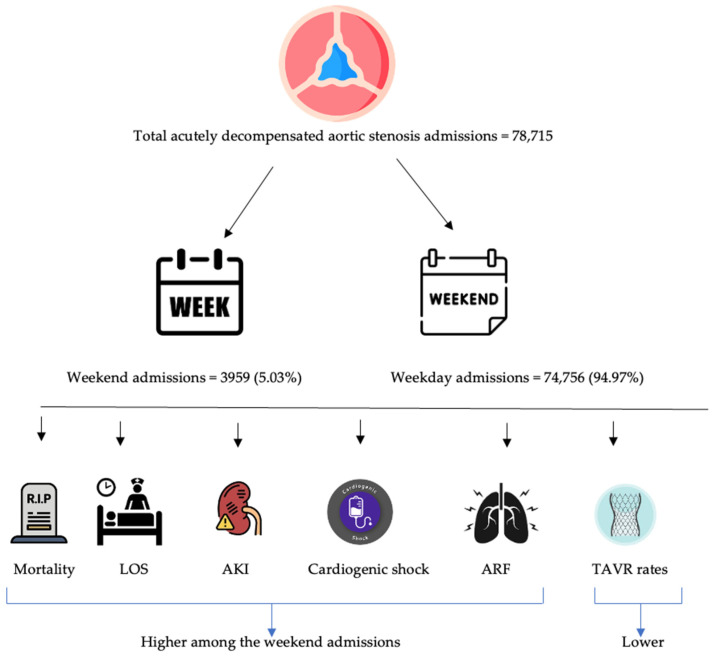
Impact of admission day on the in-hospital outcomes of patients with acutely decompensated aortic stenosis. Abbreviations: LOS—length of stay; AKI—acute kidney injury; ARF—acute respiratory failure; TAVR—transcatheter aortic valve replacement.

The NIS 2020 database, comprising 32,355,827 hospitalizations, was used for this study. Of these, 94,320 patients presented with decompensated aortic stenosis, with 4537 (4.81%) admitted on weekends and 89,783 (95.19%) admitted on weekdays. Compared to aortic stenosis patients admitted on weekdays, those admitted on weekends had higher Charlson Comorbidity Index (CCI) scores. There was no significant difference seen in age and gender distribution among the weekend and weekday admissions. However, significant differences were seen in racial distributions as Hispanics and African Americans were more likely to be admitted on weekends, whereas Caucasians were more likely to be admitted on the weekdays. Table 1 and Table 2 compares the demographic and hospital-related characteristics of aortic stenosis patients admitted on weekends versus weekdays. Figure 1 illustrates the study outcomes.

### 3.2. Primary Outcome: Mortality

**Figure 2:**
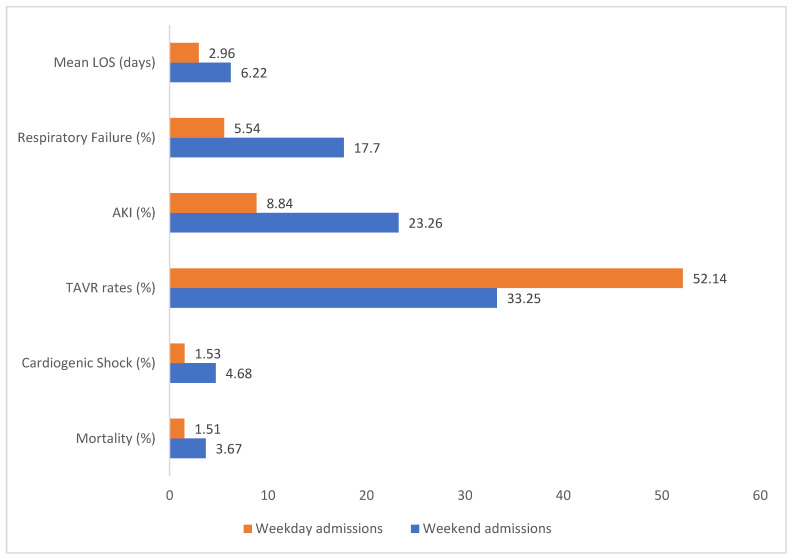
Comparison of outcomes between decompensated aortic stenosis patients admitted on weekends and weekdays. All results are significant with *p* value < 0.05.

The overall in-hospital mortality rate for patients admitted with aortic stenosis was 1.72% (1622 patients). Mortality rates were notably higher for patients admitted on weekends, at 3.42%, compared to 1.64% for those admitted on weekdays. Both univariable and multivariable analyses, adjusted for patient and hospital-level confounders, revealed that patients admitted on weekends had significantly higher odds of mortality (aOR 1.86; 95% CI 1.27–2.74) than those admitted on weekdays. Figure 2 and Table 3 illustrate a comparison of outcomes between weekend and weekday admissions.

### 3.3. Secondary Outcomes

#### 3.3.1. Resource Utilization: Length of Stay and Hospital Charges

Resource utilization was assessed by evaluating the length of stay and hospital charges. The mean hospital length of stay for patients admitted on weekends was significantly longer, with an average of 6.98 days (95% CI 6.49–7.47; *p* < 0.01), compared to 3.80 days (95% CI 3.70–3.91) for those admitted on weekdays. These results were adjusted for factors such as age, gender, race, Charlson Comorbidity Index, in-hospital mortality, and hospital teaching status. No significant differences were observed in hospital charges between weekend and weekday admissions (*p* = 0.23). Overall, these findings (Table 4) highlight the substantial strain on healthcare resources due to weekend admissions compared to weekday admissions.

#### 3.3.2. Complications

Among the complications associated with decompensated aortic stenosis, the weekend admissions, as opposed to the weekday admissions, were found to have higher rates of cardiogenic shock (5.4% vs. 2.23%) and cardiac arrest (1.54% vs. 0.91%). However, adjusted analysis only showed significantly higher odds for cardiogenic shock (aOR 2.10; 95% CI 1.54–2.87; *p* < 0.01) but not for cardiac arrest (*p* < 0.01) for the weekend vs. weekday admissions. Acute respiratory failure was more commonly seen in the weekend admissions at 18.28% (aOR 2.10; 95% CI 1.54–2.87; *p* < 0.01) compared to the weekday admissions, affecting 6.38% of cases. Similarly, acute kidney injury showed significantly higher rates (aOR 2.48; 95% CI 2.09–2.94; *p* < 0.01) for weekend admissions compared to weekday admissions (24.34% vs. 10.33%). Table 2 and Figure 2 illustrate these findings.

#### 3.3.3. Rates of Transcatheter Aortic Valve Replacement (TAVR) During the Hospitalization

Among patients admitted with decompensated aortic stenosis, TAVR was performed in 37% of the cases. The rate of TAVR was significantly lower for weekend admissions, at 25.32%, compared to 39.6% for weekday admissions (aOR 0.49; 95% CI 0.40–0.62; *p* < 0.01).

## 4. Discussion

This population-based nationwide study examined the effect of admission day on the in-hospital outcomes for patients admitted with acutely decompensated aortic stenosis. Per our knowledge and review, this is the first study of its kind using the large National Inpatient Sample in the United States. The key findings of the study indicate that patients admitted on weekends had more than double the mortality rate compared to those admitted on weekdays. Weekend admissions were also associated with longer hospital stays and higher risks of complications, including acute kidney injury, acute respiratory failure, and cardiogenic shock, compared to weekday admissions. Additionally, patients admitted on weekends had significantly lower rates of TAVR during the hospitalization compared to weekday admissions.

This study demonstrates that patients admitted with acutely decompensated aortic stenosis during weekends experience significantly worse outcomes compared to those admitted on weekdays. Weekend admissions were associated with higher mortality and morbidity, as evidenced by increased complication rates and higher resource utilization. These findings align with previous similar studies in various clinical contexts [8,9,10,11,12,13]. A large meta-analysis, which included 51,114,109 hospitalized patients, found that weekend admissions were linked to higher overall mortality [14]. This disparity in outcomes based solely on the day of admission has also been observed in other emergent cardiovascular conditions. For instance, Khoshchehreh et al. showed that patients with acute coronary syndrome admitted during weekends had higher mortality rates and lower utilization of essential invasive cardiac procedures [13]. Similar trends have been observed in cases of cardiogenic shock and cardiac arrest, where weekend admissions resulted in poorer outcomes [8,9]. Patients hospitalized with acute heart failure have also been shown to have a two-fold higher mortality risk on weekends as opposed to weekdays [10].

According to the current guidelines, aortic valve replacement is recommended for all symptomatic patients with aortic stenosis [4]. Medical therapy has not been shown to significantly alter the course or progression of the disease in these patients [15]. Over the past decade, transcatheter aortic valve replacement (TAVR) has transformed the management of aortic stenosis and is now the preferred approach for many patients [16]. Typically, TAVR is an elective procedure, involving thorough evaluation by the structural heart team, with pre-procedural valvular assessment using dedicated imaging techniques. Roule et al. demonstrated that longer wait times for TAVR, from the referral date, are independently associated with higher 1-year mortality [17]. Recently, growing evidence supports the use of urgent TAVR during the same hospitalization. In a cohort of emergently admitted patients with aortic stenosis, urgent TAVR was associated with better survival rates compared to conservative management [6]. Urgent TAVR during the same hospitalization has been shown to decrease the risk of all-cause death, cardiovascular mortality, cardiac events, and rehospitalizations within two months of admission [6]. Additionally, Abdelaziz et al. found that performing TAVR during the index hospitalization improved both clinical and echocardiographic outcomes in these patients [7].

In our cohort, TAVR was performed in 51% of admissions, with significantly lower rates among weekend admissions. Further large-scale research is needed to better understand the role of urgent TAVR in acutely decompensated aortic stenosis and to explore the significance of the variation in TAVR rates between weekend and weekday admissions. Non-elective weekend admissions for TAVR have been previously shown to be associated with higher risk of complications and increased overall risk [18]. Large-scale statistical reporting for hospital admissions has shown significantly delayed and much lower rates of procedures performed during the weekends, even when emergent [19]. This is consistent with lower TAVR rates during weekends observed in our cohort and may be the driving factor for higher mortality rates in weekend admissions for decompensated aortic stenosis. Lower staffing, discontinuity of care, and lower levels of expertise availability during weekends are the likely reasons for lower TAVR rates for these patients.

Worse outcomes for weekend admissions in decompensated aortic stenosis can be attributed to several key factors, including limitations in staffing and expertise during weekends to manage the emergent situations and complications in these patients. Additionally, the workload per healthcare professional tends to be higher during this time, which may reduce overall efficiency. Weekend teams may also have less experience working together compared to weekday teams, further impacting the quality of care. Beyond the healthcare system, patient behavior and access to care on weekends also play a role. Reduced availability of primary and social care services, particularly for vulnerable populations, can result in delayed treatment.

This “weekend effect” underscores the variability in healthcare quality between weekdays and weekends. While the day of a patient’s hospital admission is beyond their control, this factor should not affect their outcomes, especially considering the advances in modern healthcare. To mitigate this disparity, further research is required to better understand the underlying causes of the “weekend effect” and to develop strategies that ensure consistent quality of care throughout the week. Addressing known contributing factors is crucial, including optimizing the scheduling of trained staff and physicians to ensure availability on weekends without increasing workload. Incorporating physician productivity into scheduling has been shown to be an effective approach [20]. Additionally, allocating resources to hire more trained staff and physicians could provide a long-term solution, ultimately reducing resource utilization. Given the growing evidence supporting urgent TAVR, particularly in cases of severely decompensated aortic stenosis, ensuring the availability of specialist diagnostic modalities, technicians, and interventionalists on weekends may significantly improve outcomes in these patients. Large-scale, multicenter interventional studies are needed to evaluate the effectiveness of these strategies and assess their impact in reducing disparity in outcomes between weekdays and weekends.

This study has several limitations. First, as a retrospective design depending on ICD-10 codes, the study is subject to misclassification bias. Additionally, the use of an administrative database prevents accurate assessment of disease severity or co-existing conditions. We addressed this limitation by using the Charlson Comorbidity Index, a validated prognostic tool [21]. Moreover, certain key parameters, such as symptoms, lab values, the cause of aortic stenosis, and the exact reason for mortality, cannot be determined using the NIS. Despite the limitations, the large volume of administrative data in the NIS provides sufficient power to draw meaningful conclusions between the groups.

The findings from this study highlight significant disparities in outcomes based on the day of admission for patients with acutely decompensated aortic stenosis. However, being a retrospective and observational study, these findings require further validation through prospective and multicenter studies. Future studies should focus on the areas to better understand and address the causes behind the “weekend effect” observed in decompensated aortic stenosis patients. Investigating and comparing the outcomes between weekend and weekday admissions using various staffing models and allocations will play a key role. Future studies could examine the specific barriers to urgent TAVR on weekends and evaluate whether establishing protocols for expedited TAVR access or prioritizing emergency cases would improve outcomes. Weekend admissions may also suffer due to delays in obtaining diagnostic imaging, lab results, and consultations with specialists. Studies designed to analyze timelines from admission to intervention could help identify specific delays in the care pathway that disproportionately affect weekend admissions. Furthermore, understanding if certain patient demographics or comorbidity profiles are more affected by weekend admissions could help refine risk stratification and resource allocation. Lastly, policy-level research could assess the feasibility and outcomes of regulatory changes, such as incentivizing hospitals to adopt round-the-clock availability of TAVR and specialized staff, along with the cost–benefit analysis.

## 5. Conclusions

The day of admission significantly affected outcomes in patients admitted with acutely decompensated aortic stenosis, with weekend admissions associated with higher mortality compared to weekday admissions. Weekend admissions were also linked to longer hospital stays and higher rates of complications, including acute kidney injury, acute respiratory failure, and cardiogenic shock. Furthermore, patients admitted on weekends were less likely to receive urgent TAVR during the index hospitalization. These findings highlight the presence of a “weekend effect” in decompensated aortic stenosis patients, underscoring the need for targeted interventions at both the hospital and administrative levels to mitigate this impact. Implementing system-wide changes, such as increased staffing and improved resource allocation during weekends, could significantly reduce mortality and complication rates in patients admitted with acutely decompensated aortic stenosis.

## Figures and Tables

**Table 1:** Demographics characteristics of patients with decompensated aortic stenosis admitted on weekend versus weekday.

	Weekend Admission	Weekday Admission	*p* Value *
Patient characteristics			
No. (%) of patients	3959 (5.03)	74756 (94.97)	
Age (mean)	78.13 ± 0.36	77.93 ± 0.09	0.59
Female gender (%)	46.52	42.62	0.03
Race (%)			<0.01
Caucasian	78.41	86.98	
African American	7.02	4.27	
Hispanic	8.58	4.97	
Asian or Pacific islander	2.86	1.42	
Native American	0.65	0.26	
Others	2.47	2.1	
Median Income in patients Zip code (%)			0.09
USD 1–USD 47,999	26	22.01	
USD 48,000–USD 60,999	25.36	27.46	
USD 61,000–81,999	25.1	25.62	
≥USD 82,000	23.54	24.92	
Charlson comorbidity index (%)			<0.01
0	5.82	8.89	
1	14.29	21.05	
2	19.22	20.35	
3 or more	60.68	49.71	

* p value ≤ 0.05 indicates significance.

**Table 2:** Hospital characteristics of patients with decompensated aortic stenosis admitted on weekend versus weekday.

	Weekend Admission	Weekday Admission	*p* Value *
Hospital Region			0.21
Northeast	21.74	22.09	
Midwest	19.34	22.82	
South	38.18	34.99	
West	20.73	20.1	
Hospital Bed size (%)			0.01
Small	14.03	9.51	
Medium	24.65	23.53	
Large	61.31	66.96	
Hospital Location (%)			0.12
Rural	3.79	2.25	
Urban	96.21	97.75	
Hospital Teaching (%)			0.01
Non-teaching (%)	16.31	12.66	
Teaching (%)	83.69	87.34	
Insurance type (%)			<0.01
Medicaid	83.38	87.93	
Medicare	4.16	1.6	
Private	10.13	9.9	
Uninsured	2.34	0.58	

* p value ≤ 0.05 indicates significance.

**Table 3:** Impact of day of admission on patients admitted with aortic stenosis on categorical outcomes.

Outcomes	Weekend (%) (aOR; 95% CI)	Weekday (%)	*p* Value
a. Primary Outcome			
Mortality	3.67 (2.23, 1.49–3.35)	1.51 (ref)	<0.001 *
b. Secondary Outcomes			
Cardiac arrest	1.52 (1.75; 0.96–3.20)	0.82 (ref)	0.07
Cardiogenic shock	4.68 (2.76; 1.94–3.92)	1.53 (ref)	<0.001 *
Acute kidney injury	23.26 (2.48; 2.29–3.39)	8.84 (ref)	<0.001 *
Acute respiratory failure	17.7 (3.27; 2.63–4.06)	5.54 (ref)	<0.001 *
TAVR	33.25 (0.11; 0.09–0.14)	52.14 (ref)	<0.001 *
Valvuloplasty	5.44 (1.39; 0.96–1.81)	3.96 (ref)	0.09

* *p* value ≤ 0.05 indicates significance.

**Table 4:** Impact of day of admission on patients admitted with aortic stenosis on quantitative outcomes.

Outcomes	Weekend (Mean) 95% CI	Weekday (Mean) 95% CI	*p* Value
b. Secondary Outcomes			
Length of stay (days)	6.98 (6.49–7.47)	3.80 (3.69–3.91)	<0.001 *
Hospitalization charges (USD)	168,594.2 (153,019.2–184,169.3)	200,608 (192,446.3–208,769.6)	0.23

* *p* value ≤ 0.05 indicates significance.

## Data Availability

Data availability statements can be made available upon request.

## Supplementary Materials

The following supporting information can be downloaded at: https://www.mdpi.com/article/10.3390/jpm14121118/s1, Table S1: ICD 10-CM codes used for different conditions and procedures in the study. Table S2: Univariate regression coefficients for factors contributing to mortality in acutely decompensated aortic stenosis patients. Table S3: Multivariate regression coefficients for factors contributing to mortality in acutely de-compensated aortic stenosis patients.
