# Supplementary material for: Clinical Impact of Admission Day on Outcomes in Acutely Decompensated Aortic Stenosis: A Nationwide Analysis
Source: J Pers Med. 2024 Nov 25;14(12):1118. doi: 10.3390/jpm14121118 (PMC11677562; doi:10.3390/jpm14121118)
Supplement: Supplementary file 1 [file jpm-14-01118-s001.zip › jpm-3239154-supplementary.pdf]

Table S1: ICD 10-CM codes used for different conditions and procedures in the study

**Condition**	**ICD10-CM codes**
Aortic Stenosis	I060, I062, I350, I352, Q230
TAVR	02RF37H, 02RF37Z, 02RF38H, 02RF38Z, 02RF3JH, 02RF3JZ, 02RF3KH, 02RF3KZ, 02RF48Z, 02RF4JZ, 02RF4KZ, 02RF47Z
SAVR	02RF07Z, 02RF08Z, 02RF0JZ, 02RF0KZ
Acute Kidney Injury	N170, N172,N171, N178, N179
Acute Respiratory Failure	J9600, J9602, J9601, J9620, J9621, J9622, J9690, J9691,J9692
Cardiac arrest	I462, I468, I469
Cardiogenic shock	R570

Table S2: Univariate regression coefficients for factors contributing to mortality in acutely decompensated aortic stenosis patients

**Variables**	**Coefficient**	**Standard Error (SE)**	**t**	***P* value**
**Female Gender**	1.19	0.16	1.31	0.19
**Age**	1.02	0.009	2.85	0.01
**Race**				
Caucasian	ref	ref		
African American	0.88	0.29	-0.40	0.69

Hispanic	1.13	0.30	0.46	0.65
Asian or Pacific islander	0.76	0.43	-0.48	0.63
Native American	1.33	1.38	0.28	0.78
Others	0.37	0.27	-1.37	0.17
**Median Income in patients Zip code**				
$1-$47,999	ref	ref		
$48,000-$60,999	0.85	0.15	-0.92	0.36
$61,000-81,999	0.85	0.15	-0.90	0.37
≥$82,000	0.59	0.12	-2.64	0.008
**Charlson comorbidity index**				
**Hospital Region**				
Northeast	ref	ref		
Midwest	1.20	0.27	0.80	0.43
South	1.18	0.24	0.79	0.43
West	0.96	0.21	-0.16	0.87
**Hospital Teaching Status**	1.34	0.28	1.49	0.14
**Hospital Location**	1.24	0.55	0.49	0.63
**Hospital Bed Size**				
Small	ref	ref		
Medium	0.74	0.17	-1.31	0.19
Large	0.78	0.15	-1.28	0.20

Table S3: Multivariate regression coefficients for factors contributing to mortality in acutely decompensated aortic stenosis patients

**Variables**	**Coefficient**	**Standard Error (SE)**	t	*P* value

**Female Gender**	1.28	0.17	1.87	0.06
**Age**	1.03	0.01	2.91	0.01
**Race**				
Caucasian	ref	ref		
African American	0.67	0.22	-1.21	0.23
Hispanic	1.03	0.28	0.10	0.92
Asian or Pacific islander	0.71	0.41	-0.60	0.55
Native American	1	na	na	na
Others	0.38	0.28	-1.32	0.19
**Median Income in patients Zip code**				
$1-$47,999	ref	ref		
$48,000-$60,999	0.87	0.17	-0.71	0.48
$61,000-81,999	0.81	0.16	-1.06	0.29
≥$82,000	0.62	0.13	-2.25	0.02
**Charlson comorbidity index**	1.31	0.03	10.56	<0.01
**Hospital Region**				
Northeast	ref	ref		
Midwest	1.05	0.25	0.21	0.84
South	1.10	0.24	0.46	0.65
West	0.93	0.21	-0.34	0.73
**Hospital Teaching Status**	1.55	0.37	1.83	0.07
**Hospital Location**	1.17	0.63	0.30	0.77
**Hospital Bed Size**				
Small	ref	ref		
Medium	0.71	0.17	-1.42	0.15
Large	0.78	0.16	-1.24	0.22
